# Identifying Druggable Inflammatory Proteins Causally Contributing to Parkinson's Disease

**DOI:** 10.1002/brb3.70992

**Published:** 2025-10-20

**Authors:** Ziyao Zhang, Hongbao Cao, Ancha Baranova, Fuquan Zhang

**Affiliations:** ^1^ Department of Psychiatry The Affiliated Brain Hospital of Nanjing Medical University Nanjing China; ^2^ School of Systems Biology George Mason University Manassas Virginia USA; ^3^ Research Centre for Medical Genetics Moscow Russia

**Keywords:** circulating inflammatory proteins, Gene‐Drug Interaction Analysis, GWAS, Mendelian randomization, Parkinson's disease

## Abstract

**Background:**

Inflammatory mechanisms are critically engaged in the pathogenesis of Parkinson's disease (PD). The objective of this research was to detect circulating inflammatory proteins that potentially elevate the susceptibility to PD.

**Methods:**

A two‐sample Mendelian randomization (MR) analysis was performed to determine the causal effects of 91 circulating inflammatory proteins (14,824 participants) on PD (33,674 cases and 449,056 controls) by using genome‐wide association study (GWAS) summary data. The MR analysis utilized three complementary approaches: inverse variance weighted (IVW), weighted median, and MR‐Egger. Among them, IVW was the principal method. Additionally, we utilized the Drug‐Gene Interaction Database (DGIdb) to select potential therapeutic targets for proteins associated with PD.

**Results:**

Our MR analysis revealed that six circulating inflammatory proteins were associated with PD. Three of them conferred a protective effect against PD, including TNFRSF9 (odds ratio [OR]: 0.83, 95% confidence interval [CI]: 0.74–0.92, *p* = 5.09 × 10^−4^), Flt3L (OR: 0.89, 95% CI: 0.81–0.99, *p* = 0.026), and TGF‐α (OR: 0.86, 95% CI: 0.75–0.99, *p* = 0.04). Three additional proteins were connected with an increased risk of PD, namely, IL‐18 (OR: 1.13, 95% CI: 1.02–1.24, *p* = 0.016), CD6 (OR: 1.08, 95% CI: 1.01–1.16, *p* = 0.023), and IL‐17A (OR: 1.18, 95% CI: 1.01–1.38, *p* = 0.033).

**Conclusion:**

Our research revealed the pathogenic contribution of several inflammatory proteins to PD development, providing new perspectives on the mechanisms and therapy of PD.

## Graphical abstract

Six circulating inflammatory proteins affect Parkinson's disease risk. IL‐18 and IL‐17A are potential therapeutic targets, offering new insights for mechanisms and therapy.

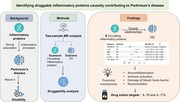

## 1 Introduction

Parkinson's disease (PD) is a progressive neurological disorder (Jankovic 2008), characterized by core motor symptoms such as bradykinesia, muscular rigidity, resting tremor, and gait abnormalities (Parkinson 2002). The pathogenesis of PD remains incompletely understood but is known to arise from the interaction between genetic susceptibility and environmental factors. The main pathological manifestation of PD is neurodegeneration in brain regions such as the substantia nigra due to the aggregation of alpha‐synuclein (Dickson 2018). Some studies have shown that the number of PD patients doubled to over 6 million from 1990 to 2015, and it is projected to exceed 12 million cases by 2040 (Dorsey et al. 2018). The rising development trend of PD compels us to pay great attention to this disease and spare no effort in searching for effective therapeutic solutions to deal with this severe health challenge.

Numerous studies have shown that inflammation has a large impact on the pathogenesis of PD (Kline et al. 2021). From disease initiation, inflammatory processes start centrally and peripherally in PD, remaining persistent during pathological progression (Joshi and Singh 2018). Inflammation begins with the activation of microglia in central nervous system (CNS) compartments (Gundersen 2020). When alpha‐synuclein stimulates resting microglia, it activates these cells and triggers the release of pro‐inflammatory cytokines such as interleukins (ILs) (Q. S. Zhang et al. 2017), initiating a cascade of inflammatory responses that ultimately lead to the death of dopaminergic neurons (Tan et al. 2020). In addition to this, some studies point out that immune cells within the peripheral circulation of PD patients produce larger quantities of inflammatory agents like IL‐6 and IL‐1b (Qin et al. 2016), suggesting that inflammation activation also occurs within the peripheral tissue systems of PD patients. The compromised blood‐brain barrier (BBB) enables immunocytes originating in the peripheral bloodstream to penetrate the parenchyma of the brain (Yi et al. 2023), ultimately leading to dopaminergic neuronal degeneration and the development of PD. Therefore, as an indispensable component in the progression of inflammation, it is suggested that inflammatory factors contribute to the emergence and advancement of PD (Y. Zhao et al. 2022).

Recently, increasing numbers of studies have begun to investigate the pathogenesis of PD from the perspective of genetics. In this regard, genome‐wide association studies (GWAS) offer a hypothesis‐free way to investigate a large number of genetic variants in human genomes and have been widely used in many common and complex diseases (Rhodes et al. 2011). Studying the genetic markers and risk factors of PD identified through GWAS can help us understand the pathological process of PD (Kia et al. 2021).

As an analytical method, Mendelian randomization (MR) serves to assess the cause‐and‐effect association among risk factors and clinical outcomes (Sekula et al. 2016). By employing genetic variants as instrumental variables (IVs), MR analysis enables causal inference between exposure phenotypes and clinical outcomes. MR has been widely employed to deduce the link between exposure and outcome (Baranova et al. 2024; Cao et al. 2024; D. Liu et al. 2024; Q. Zhao, Liu, et al. 2024). During our research, we used two‐sample MR analysis to investigate the cause‐and‐effect linkages between the levels of 91 circulating inflammatory proteins and PD Table S1.

## 2 Methods

### 2.1 GWAS Summary Datasets

The GWAS summary data adopted for this study were publicly available. All subjects were of European ancestry, and all original studies were ethically approved. The outcome dataset (33,674 cases and 449,056 controls) was sourced from a PD GWAS (https://bit.ly/2ofzGrk) carried out by the International Parkinson's Disease Genomics Consortium (IPDGC), which included three GWAS investigations reported earlier, 13 recently acquired datasets, alongside UKB proxy‐case data (leaving out 23andMe) (Nalls et al. 2019). Additionally, data on 91 circulating inflammatory proteins (https://www.phpc.cam.ac.uk/ceu/proteins/ and https://www.ebi.ac.uk/gwas/ [accession GCST9027065‐GCST90270855]) originated from 11 cohorts with a total of 14,824 participants (J. H. Zhao et al. 2023). Building on previous research, this GWAS conducted pQTL mapping for 91 circulating inflammatory proteins among 14,824 participants (J. H. Zhao et al. 2023).

### 2.2 MR Analysis

**FIGURE 1:**
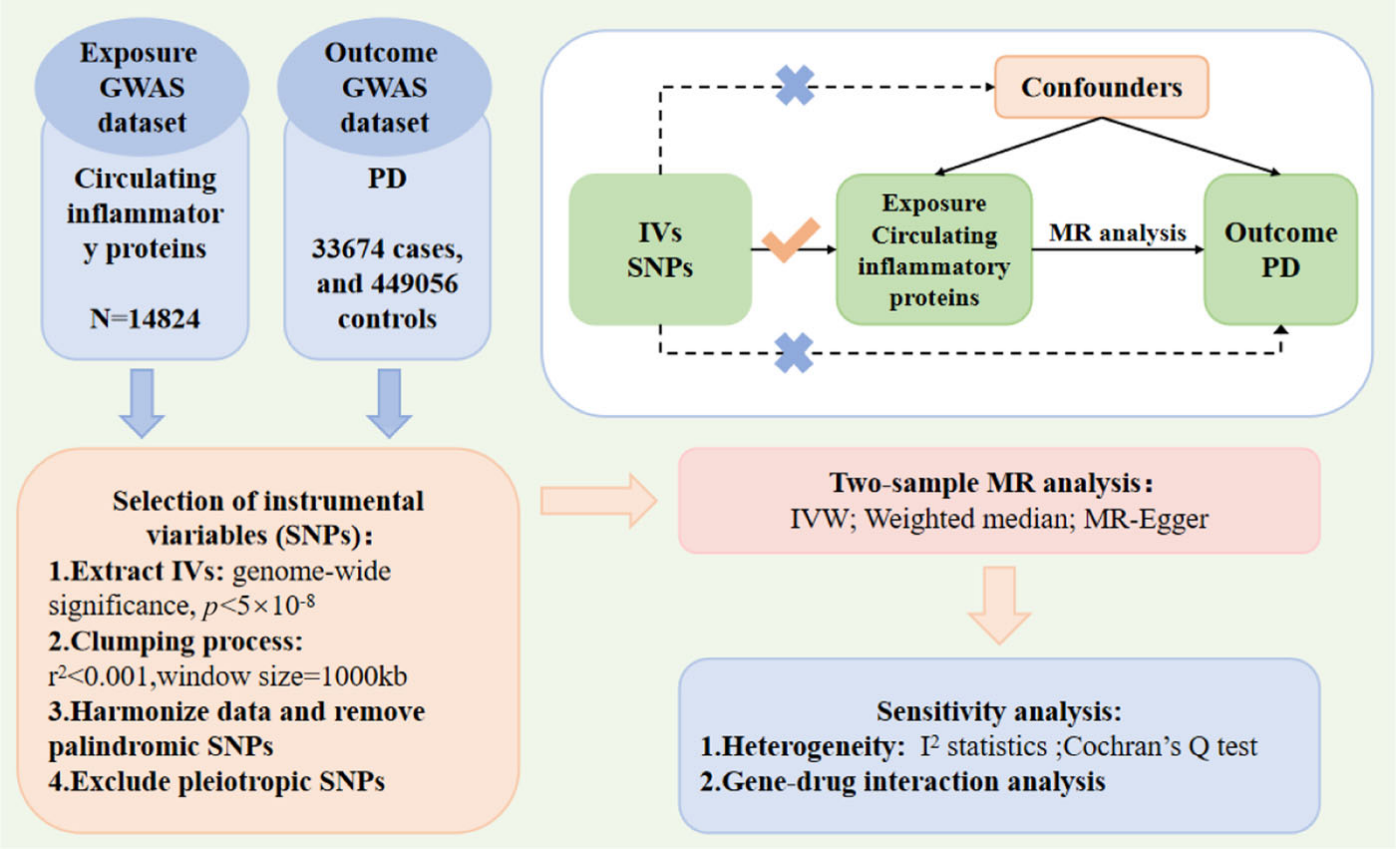
Study workflow. GWAS: genome‐wide association studies, IVW: inverse variance weighted, PD: Parkinson's disease, SNPs: single‐nucleotide polymorphisms.

In R (version 4.4.1), we carried out the MR on the circulating inflammatory proteins alongside PD following the process steps shown in Figure 1. When performing this analysis, we integrated three complementary methods into the TwoSampleMR (version 0.5.6) (Hemani et al. 2018): inverse variance weighted (IVW), weighted median (WM), and MR‐Egger. Three core assumptions must be met by single‐nucleotide polymorphisms (SNPs) serving as IVs in two‐sample MR studies (Jankovic 2008): IVs are highly related to exposure (Parkinson 2002), there is no connection between IVs and any confounding factors (Dickson 2018), and IVs impact the outcome only via the exposure (Cheng et al. 2023). The IVW was our principal method (Bowden et al. 2015). The intercept is assumed to be zero, and consistent causality estimates are provided by a fixed‐effects meta‐analysis. In addition, we adopted the WM and MR‐Egger as two supplementary ways to measure sensitivity. Starting from the fundamental assumption that pleiotropic effects are independent, the MR‐Egger framework conducts a weighted linear regression to explore relationships between exposure and outcome coefficients (Bowden et al. 2015). We used *I* ^2^ statistics and Cochran's *Q* test to assess horizontal pleiotropy. Both *I* ^2^ statistics and Cochran's *Q* test were utilized to measure the heterogeneity. If the *p*‐value falls below 0.05 and the *I* ^2^ value exceeds 0.25, the results are determined to have significant heterogeneity. If the significance level of the IVW method was *p* < 0.05, a causal link between the circulating inflammatory proteins and PD was considered to exist.

For the MR analysis, we obtained statistics from the 1000 Genomes Project Phase 3 (EUR) and selected SNPs that met genome‐wide significance (*p* < 5 × 10^−^⁸) to serve as IVs. The pruning process applied a clumping *r* ^2^ cutoff of 0.001 within a 10 Mb window. If the number of IVs was small, we used a comparatively lenient threshold of 1 × 10^−^⁵ for IV selection. When performing TSMR analysis, we ensured that SNPs influencing both exposure and outcomes acted on the same alleles by using the “harmonise_data” function and aligning the effect alleles between exposure and outcome datasets. To maintain the accuracy and ensure the reliability of the results, we removed SNPs absent from the outcome dataset and palindromic SNPs with intermediate allele frequencies (minor allele frequency ≥ 0.42) (Zhao, Baranova, et al. 2024).

### 2.3 Gene‐Drug Interaction Analysis

We utilized the Drug‐Gene Interaction Database (DGIdb) (https://old.dgidb.org/) to examine the genes related to inflammatory proteins associated with PD identified by MR analysis. Subsequently, we identified certain drug‐acting targets.

## 3 Results

### 3.1 MR Analysis

**FIGURE 2:**
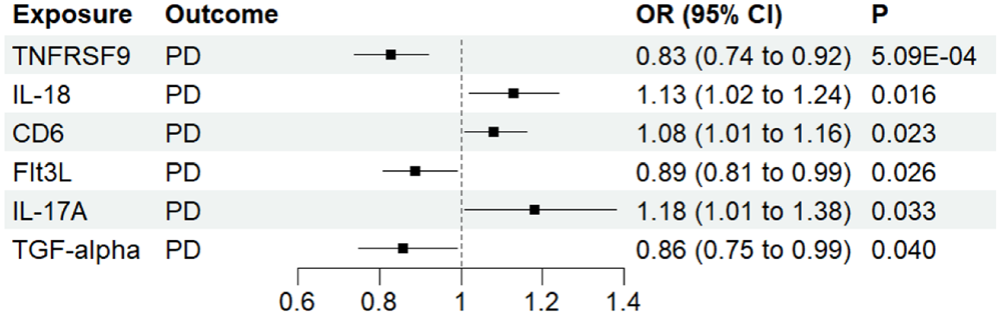
Causal effects of the circulating inflammatory proteins on PD. CI: confidence interval, OR: odds ratio, PD: Parkinson's disease.

**FIGURE 3:**
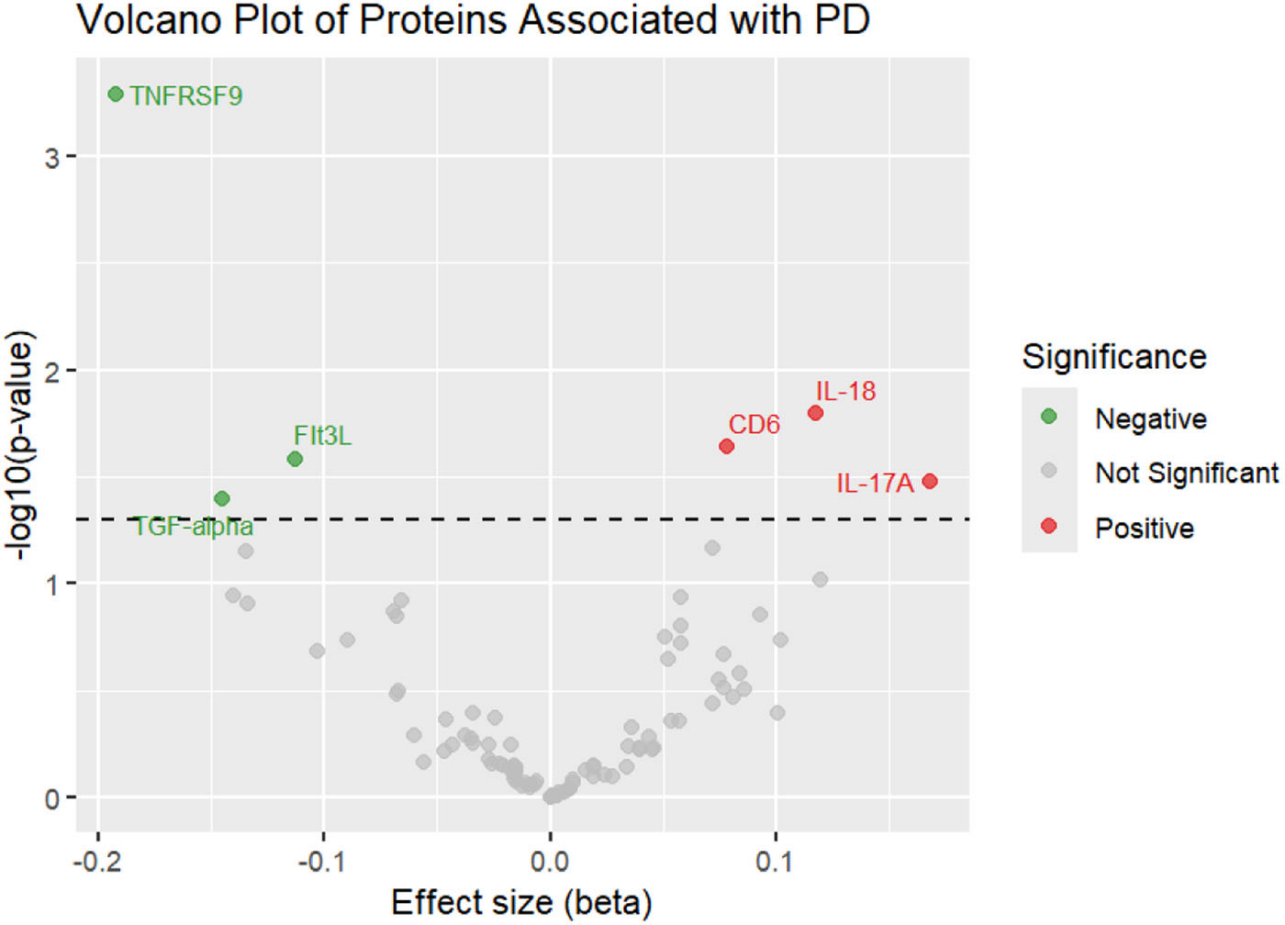
Volcano plot of the causal effects between circulating inflammatory proteins and PD. Significant proteins are indicated by color (red for positive, gray for not significant, and green for negative).

As shown in Table 1, this MR analysis uncovered six circulating inflammatory proteins that were significantly causally associated with PD. Three proteins exert a protective effect on PD, including TNFRSF9 (odds ratio [OR]: 0.83, 95% confidence interval [CI]: 0.74–0.92, *p* = 5.09E‐04), Flt3L (OR: 0.89, 95% CI: 0.81–0.99, *p* = 0.026), and TGF‐α (OR: 0.86, 95% CI: 0.75–0.99, *p* = 0.04) (Figure 2). On the contrary, IL‐18 (OR: 1.13, 95% CI: 1.02–1.24, *p* = 0.016), CD6 (OR: 1.08, 95% CI: 1.01–1.16, *p* = 0.023), and IL‐17A (OR: 1.18, 95% CI: 1.01–1.38, *p* = 0.033) contributed to the PD risk. The results of the MR‐Egger and the WM techniques yielded results similar to those of the main model. However, only TNFRSF9 remained significant after false discovery rate (FDR) correction (FDR < 0.05). Figure 3 presents a volcano plot depicting the relative strength of the causal relationship between PD and circulating inflammatory proteins. MR sensitivity analysis indicated consistent directions of causal effect estimates across applied techniques. Results from the MR‐Egger model in the sensitivity analysis indicated the absence of significant horizontal pleiotropy (p > 0.05). In addition, we conducted MR‐PRESSO analysis, and the results also showed no horizontal pleiotropy. Meanwhile, Cochrane's *Q* test and *I* ^2^ statistics showed that no heterogeneity was present in the MR estimates (*p* > 0.05).

**TABLE 1:** Causal effects of the circulating inflammatory proteins on PD.

Exposure	Outcome	*B* (SE)	OR [95%CI]	Q_P	*I* ^2^	P_pleiotropy	*p*	FDR
TNFRSF9	PD	−0.192 (0.055)	0.83 [0.74–0.92]	0.625	−0.108	0.276	5.09E‐04	0.046
IL‐18	PD	0.118 (0.049)	1.13 [1.02–1.24]	0.502	−0.023	0.943	0.016	0.728
CD6	PD	0.078 (0.035)	1.08 [1.01–1.16]	0.368	0.068	0.462	0.023	0.698
Flt3L	PD	−0.113 (0.051)	0.89 [0.81–0.99]	0.132	0.193	0.476	0.026	0.591
IL‐17A	PD	0.168 (0.079)	1.18 [1.01–1.38]	0.956	−0.854	0.75	0.033	0.601
TGF‐α	PD	−0.145 (0.071)	0.86 [0.75–0.99]	0.543	−0.058	0.383	0.040	0607

Abbreviations: *B* effect size, CI: confidence interval, FDR: false discovery rate, N_IV: number of instrumental variables, OR: odds ratio, PD: Parkinson's disease, Q_P: Cochran's *p* value of heterogeneity analysis, SE: standard error.

### 3.2 Gene‐Drug Interaction Analysis

By mining the DGIdb database, we found that IL‐18 and IL‐17A cytokines are targeted by multiple existing drugs (Table 2). However, these drugs are either monoclonal antibodies that cannot penetrate the BBB and, therefore, are used for the treatment of PD, or their safety and efficacy remain unclear. Therefore, our analysis of druggability should be regarded as a support for a certain hypothesis rather than a direct clinical recommendation. More studies are warranted to verify the biological mechanisms connecting particular soluble cytokines and the risks of PD, and to define the feasibility of the manipulation of their levels for alleviating PD phenotypes.

**TABLE 2:** The results of the gene drug interaction.

Gene	Drug	Regulatory approval	Indication	Interaction score
IL18	Mycophenolate	Approved	Immunosuppressant	0.24
IL18	Colchicine	Approved	For the treatment of gout	0.1
IL18	Anhydrous tacrolimus	Approved	Immunosuppressant	0.19
IL18	Peginterferon alfa‐2B	Approved	NA	0.17
IL18	Peginterferon alfa‐2A	Approved	Antineoplastic agents and immunomodulatory agents, for the treatment of hepatitis B and C	0.18
IL18	Interferon alfa‐2B	Approved	For the treatment of hepatitis C, antineoplastic agents; immunomodulatory agents, for the treatment of hepatitis B and C, and for the treatment of hepatitis	0.39
IL18	Ribavirin	Approved	NA	0.14
IL‐17A	Simvastatin	Approved	Antidyslipidemic agent, anticholesterolemic agent, antihypertensive agent	0.8
IL‐17A	Secukinumab	Approved	NA	2
IL‐17A	Bimekizumab	Approved	NA	2
IL‐17A	Sirolimus	Approved	For the treatment of wet age‐related macular degeneration, an immunosuppressant	0.06
IL‐17A	Gentamicin	Approved	NA	0.17
IL‐17A	Azathioprine	Approved	NA	0.1
IL‐17A	Brodalumab	Approved	NA	0.66
IL‐17A	Ixekizumab	Approved	NA	4.01
IL‐17A	Tetracycline	Approved	NA	0.22
IL‐17A	Erythromycin	Approved	NA	0.17
IL‐17A	Mannitol	Approved	NA	0.66

Abbreviation: NA: not applicable.

## 4 Discussion

The causes of PD are multifaceted, involving both social and genetic factors. Previous studies have indicated an association between immune activation and certain inflammatory biomarkers with the pathophysiological changes in PD (Adamowicz et al. 2024; Patlola et al. 2023). Our research indicates that particular circulating inflammatory proteins may either promote or suppress the development of PD. In particular, three protective proteins and three risk proteins for PD were found.

Our results show that the levels of IL‐18 are positively correlated with the risk of PD. As a key pro‐inflammatory cytokine belonging to the interleukin‐1 (IL‐1) superfamily, IL‐18 significantly contributes to neuroinflammation and neurodegeneration (X. Xu et al. 2011; Y. Xu et al. 2016). Multiple studies have suggested that IL‐18 is abnormally expressed in various cohorts of patients with mental disorders and is usually regarded as a potential biomarker reflecting the deteriorating condition of the brain (Y. Xu et al. 2016; Chen et al. 2024; Hayek et al. 2024; Lin et al. 2023; Sun et al. 2025; Zheng et al. 2024). The levels of IL‐18 in the serum of patients with PD are significantly elevated (Li et al. 2021). Additionally, certain polymorphisms in the promoter of the IL‐18 encoding gene raise the likelihood of developing PD (Fahmy et al. 2019). IL‐18 directly promotes microglial activation, which is accompanied by enhancement of dopaminergic excitotoxicity (Lang et al. 2018), which is one of the well‐recognized mechanisms for the promotion of PD symptoms. Some studied compounds, such as JNK‐activating flavonol juglanin found in *Polygonum aviculare*, ameliorate neurodegeneration and delay the onset of PD by inhibiting the production of pro‐inflammatory factors, including IL‐18 (F. X. Zhang and Xu 2018).

Another risk factor for PD, IL‐17A, was highlighted as pathophysiologically relevant to neurodegeneration in a variety of previous studies. In particular, significantly elevated levels of IL‐17A were reported in both cerebrospinal fluid (CSF) and plasma samples collected from PD patients, and correlated with disease severity and progression (Gu et al. 2013). Production of Interleukin‐17A (IL‐17A) is a characteristic feature of the CD4+ helper T cells subgroup known as Th17 cells (McGeachy et al. 2019). IL‐17A contributes to the pathogenesis and progression of various neurodegenerative disorders of the CNS. In PD, it stimulates microglia by triggering neuroinflammation and neurodegenerative changes associated with the development of PD phenotypes (Kolbinger et al. 2016; Zou et al. 2023). In drug‐gene interaction analysis, we identified IL‐17A as a potential therapeutic target. In consistency with this finding, anti‐IL‐17A neutralizing antibodies effectively alleviate PD symptoms in a rat model (Z. Liu et al. 2019). Moreover, reduction of IL‐17A production represents a mediating factor connecting repetitive transcranial magnetic stimulation (rTMS) and mitigation of neuroinflammation (Aftanas et al. 2018).

This study also found that the levels of CD6 are positively correlated with the risks of PD. Surface molecule CD6 decorates T cells and participates in T cell regulation (Gonçalves et al. 2018). An increasing number of studies have confirmed that T cells play an important role in PD (Contaldi et al. 2022). In particular, in PD patients, affected brain regions are characterized by extensive T‐cell infiltrates, which may contribute to the disruption of the BBB (Garretti et al. 2022) and subsequent degeneration of other parts of the CNS (Sæther et al. 2023). It is plausible to hypothesize that CD6 may cause an increase in the risks of PD by altering T‐cell characteristics and, therefore, promoting the disruption of the BBB.

TGF‐α is produced by neurons and glial cells of the CNS (Ferrer et al. 1995). In vitro, this cytokine exerts a nutritive effect on dopaminergic neurons in the midbrain (Engele and Schilling 1996). Interestingly, some studies of the brain and CSF samples of PD patients demonstrate elevation in TGF‐α levels (Mogi et al. 1994), an observation that contradicts the results of the current study. We speculate that an increase in plasma levels of TGF‐α may represent a compensatory mechanism and is secondary to the extent of neurodegeneration (Connor and Dragunow 1998), rather than being a primary pathogenetic force. In fact, some studies point out that TGF‐α may promote the generation of new dopaminergic neurons and may serve as an indicator for assessing the efficacy of dopaminergic cell transplantation into the substantia nigra of patients with PD (Blum 1998). Obviously, the mechanisms of TGF‐α effects exerted upon PD phenotypes are far from being clear and warrant further investigation.

In consistency with previous studies (Herbert et al. 2015), our study reports a negative correlation between the levels of Flt3L in the serum and the risks of PD. Flt3L is a hematopoietic cytokine found in a variety of tissues, including the brain (Brazel et al. 2001). While this molecule is crucial for the survival of hematopoietic stem cells (Shurin 1998) and capable of accelerating the differentiation of macrophages, dendritic cells, and microglia (Servet‐Delprat et al. 2002), it also possesses neurotrophic properties (Brazel et al. 2001). Potential protective mechanisms connecting the elevation of Flt3L and the risks of PD remain to be elucidated.

TNFRSF9 was the only protein with levels that remained significantly associated with PD risk after FDR correction. MR results indicated that TNFRSF9 is a protective factor against the risks of PD. As a member of the tumor necrosis factor receptor (TNFR) superfamily, it is produced in activated antigen‐presenting cells and can induce survival signals in T cells (Lee et al. 2005). Notably, at least some cases of PD may be associated with the somatic loss of the TNFRSF9‐encoding gene (Güler et al. 2021). Levels of TNFRSF9 expression negatively correlate with PD severity (Hepp et al. 2023). Although its role in PD remains elusive, its expression in neurons and astrocytes indicates potential involvement in the control of neuroinflammation in the CNS (Reali et al. 2003; Yeo et al. 2012). It may enhance the immunosuppressive functions of T cells, with the specific mechanisms awaiting experimental dissection.

MR analysis is a powerful tool that can overcome the problems posed by confounding effects and reverse causation. Therefore, it greatly reduces the interference in the analysis of findings in classical epidemiological investigations. However, there remain certain limitations in our research. First, only TNFRSF9 remained significantly associated with PD risk after FDR correction. Second, although our sensitivity analyses (such as MR‐Egger and WM methods) did not find significant pleiotropy, undetected hidden pleiotropic pathways may still exist, which could introduce bias into the results. All of our data are from European populations, which limits population representativeness. Our findings may not be directly generalized to non‐European populations. Furthermore, although we implemented stringent IV selection criteria (*p* < 5 × 10^−^⁸) and removed SNPs in linkage disequilibrium (*r* ^2^ < 0.001), the potential influence of weak instruments cannot be completely ruled out. Consequently, the results should be interpreted cautiously. At the same time, we should consider the influence of canalization, which may mask the true association between these proteins and PD. Finally, although we have identified inflammatory proteins related to PD, their biological mechanisms remain unclear and require further research for verification.

## 5 Conclusion

The study identified three risk inflammatory proteins and three that play a protective role during the progression of PD, offering novel perspectives on the mechanisms and possible avenues for the treatment of this disease.

## Author Contributions

**Ziyao Zhang**: writing – review and editing, writing – original draft, visualization. **Ancha Baranova**: validation, writing – review and editing, writing – original draft. **Hongbao Cao**: validation, writing – review and editing, writing – original draft. **Fuquan Zhang**: formal analysis, writing – review and editing, writing – original draft, supervision, conceptualization, investigation, data curation, project administration.

## Funding Information

The authors received no specific funding for this work.

## Peer Review

The peer review history for this article is available at https://publons.com/publon/10.1002/brb3.70992

## Supporting information




**Supplementary Table**: brb370992‐sup‐0001‐TableS1.xlsx

## Data Availability

All GWAS summary datasets in this study are publicly available for download by qualified researchers.

## References

[brb370992-bib-0001] Adamowicz, D. H. , T.‐C. Wu , R. Daly , et al. 2024. “Executive Functioning Trajectories and Their Prospective Association With Inflammatory Biomarkers in Schizophrenia and Non‐Psychiatric Comparison Participants.” Progress in Neuro‐Psychopharmacology & Biological Psychiatry 128: 110866. 10.1016/j.pnpbp.2023.110866. 37742747 PMC10829566

[brb370992-bib-0002] Aftanas, L. I. , M. M. Gevorgyan , S. Y. A. Zhanaeva , et al. 2018. “Therapeutic Effects of Repetitive Transcranial Magnetic Stimulation (rTMS) on Neuroinflammation and Neuroplasticity in Patients With Parkinson's Disease: A Placebo‐Controlled Study.” Bulletin of Experimental Biology and Medicine 165: 195–199. 10.1007/s10517-018-4128-4. 29923005

[brb370992-bib-0003] Baranova, A. , H. Cao , and F. Zhang . 2024. “Exploring the Influences of Education, Intelligence and Income on Mental Disorders.” General Psychiatry 37: e101080. 10.1136/gpsych-2023-101080. 38440407 PMC10910399

[brb370992-bib-0004] Blum, M. 1998. “A Null Mutation in TGF‐Alpha Leads to a Reduction in Midbrain Dopaminergic Neurons in the Substantia Nigra.” Nature Neuroscience 1: 374–377. 10.1038/1584. 10196526

[brb370992-bib-0005] Bowden, J. , G. Davey Smith , and S. Burgess . 2015. “Mendelian Randomization With Invalid Instruments: Effect Estimation and Bias Detection Through Egger Regression.” International Journal of Epidemiology 44: 512–525. 10.1093/ije/dyv080. 26050253 PMC4469799

[brb370992-bib-0006] Brazel, C. Y. , M. H. Ducceschi , B. Pytowski , and S. W. Levison . 2001. “The FLT3 Tyrosine Kinase Receptor Inhibits Neural Stem/Progenitor Cell Proliferation and Collaborates With NGF to Promote Neuronal Survival.” Molecular and Cellular Neuroscience 18: 381–393. 10.1006/mcne.2001.1033. 11640895

[brb370992-bib-0007] Cao, H. , L. Fu , D. Liu , A. Baranova , and F. Zhang . 2024. “Mendelian Randomization Analysis of Causal and Druggable Circulating Inflammatory Proteins in Schizophrenia.” Frontiers in Psychiatry 15: 1465291. 10.3389/fpsyt.2024.1465291. 39544374 PMC11560794

[brb370992-bib-0008] Chen, W. , Y. Tian , M. Gou , et al. 2024. “Role of the Immune‐Kynurenine Pathway in Treatment‐Resistant Schizophrenia.” Progress in Neuro‐Psychopharmacology & Biological Psychiatry 130: 110926. 10.1016/j.pnpbp.2023.110926. 38147973

[brb370992-bib-0009] Cheng, W. , Y. Liao , R. Mou , X. Xiao , and Y. Jia . 2023. “Inflammatory Bowel Disease and Prostate Cancer Risk: A Two‐Sample Mendelian Randomization Analysis.” Frontiers in Immunology 14: 1157313. 10.3389/fimmu.2023.1157313. 37409117 PMC10318899

[brb370992-bib-0010] Connor, B. , and M. Dragunow . 1998. “The Role of Neuronal Growth Factors in Neurodegenerative Disorders of the Human Brain.” Brain Research Reviews 27: 1–39. 10.1016/S0165-0173(98)00004-6. 9639663

[brb370992-bib-0011] Contaldi, E. , L. Magistrelli , and C. Comi . 2022. “T Lymphocytes in Parkinson's Disease.” Journal of Parkinson's Disease 12: S65–S74. 10.3233/JPD-223152. PMC9535550 35253782

[brb370992-bib-0012] Dickson, D. W. 2018. “Neuropathology of Parkinson Disease.” Parkinsonism & Related Disorders 46, no. 1: S30–S33. 10.1016/j.parkreldis.2017.07.033. 28780180 PMC5718208

[brb370992-bib-0013] Dorsey, E. R. , T. Sherer , M. S. Okun , and B. R. Bloem . 2018. “The Emerging Evidence of the Parkinson Pandemic.” Journal of Parkinson's Disease 8: S3–S8. 10.3233/JPD-181474. PMC6311367 30584159

[brb370992-bib-0014] Engele, J. , and K. Schilling . 1996. “Growth Factor‐Induced C‐Fos Expression Defines Distinct Subsets of Midbrain Dopaminergic Neurons.” Neuroscience 73: 397–406. 10.1016/0306-4522(96)00045-0. 8783257

[brb370992-bib-0015] Fahmy, E. , A. Rabah , S. Sharaf , H. Helmy , and A. Kamal . 2019. “Interleukin‐18 Promoter Polymorphisms and Idiopathic Parkinson Disease: An Egyptian Study.” Acta Neurologica Belgica 119: 219–224. 10.1007/s13760-018-0927-7. 29700781

[brb370992-bib-0016] Ferrer, I. , R. Blanco , M. Carulla , et al. 1995. “Transforming Growth Factor‐Alpha Immunoreactivity in the Developing and Adult Brain.” Neuroscience 66: 189–199. 10.1016/0306-4522(94)00584-R. 7637868

[brb370992-bib-0017] Garretti, F. , C. Monahan , A. Sette , D. Agalliu , and D. Sulzer . 2022. “T Cells, α‐Synuclein and Parkinson Disease.” Handbook of Clinical Neurology 184: 439–455. 35034753 10.1016/B978-0-12-819410-2.00023-0 PMC10193709

[brb370992-bib-0018] Gonçalves, C. M. , S. N. Henriques , R. F. Santos , and A. M. Carmo . 2018. “CD6, a Rheostat‐Type Signalosome That Tunes T Cell Activation.” Frontiers in Immunology 9: 2994. 10.3389/fimmu.2018.02994. 30619347 PMC6305463

[brb370992-bib-0019] Gu, C. , L. Wu , and X. Li . 2013. “IL‐17 Family: Cytokines, Receptors and Signaling.” Cytokine 64: 477–485. 10.1016/j.cyto.2013.07.022. 24011563 PMC3867811

[brb370992-bib-0020] Güler, S. , T. Gül , Ş. Güler , M. C. Haerle , and A. N. Başak . 2021. “Early‐Onset Parkinson's Disease: A Novel Deletion Comprising the DJ‐1 and TNFRSF9 Genes.” Movement Disorders 36: 2973–2976. 34605055 10.1002/mds.28812

[brb370992-bib-0021] Gundersen, V. 2020. “Parkinson's Disease: Can Targeting Inflammation Be an Effective Neuroprotective Strategy?.” Frontiers in Neuroscience 14: 580311. 10.3389/fnins.2020.580311. 33716638 PMC7946840

[brb370992-bib-0022] Hayek, D. , G. Ziegler , L. Kleineidam , et al. 2024. “Different Inflammatory Signatures Based on CSF Biomarkers Relate to Preserved or Diminished Brain Structure and Cognition.” Molecular Psychiatry 29: 992–1004. 10.1038/s41380-023-02387-3. 38216727 PMC11176056

[brb370992-bib-0023] Hemani, G. , J. Zheng , B. Elsworth , et al. 2018. “The MR‐Base Platform Supports Systematic Causal Inference Across the Human Phenome.” Elife 7: e34408. 10.7554/eLife.34408. 29846171 PMC5976434

[brb370992-bib-0024] Hepp, D. H. , T. A. Van Wageningen , K. L. Kuiper , et al. 2023. “Inflammatory Blood Biomarkers Are Associated With Long‐Term Clinical Disease Severity in Parkinson's Disease.” International Journal of Molecular Sciences 24: 14915. 10.3390/ijms241914915. 37834363 PMC10573398

[brb370992-bib-0025] Herbert, M. K. , M. B. Aerts , M. Beenes , et al. 2015. “CSF Neurofilament Light Chain but Not FLT3 Ligand Discriminates Parkinsonian Disorders.” Frontiers in Neurology 6: 91. 10.3389/fneur.2015.00091. 25999911 PMC4419719

[brb370992-bib-0026] Jankovic, J. 2008. “Parkinson's Disease: Clinical Features and Diagnosis.” Journal of Neurology, Neurosurgery, and Psychiatry 79: 368–376. 10.1136/jnnp.2007.131045. 18344392

[brb370992-bib-0027] Joshi, N. , and S. Singh . 2018. “Updates on Immunity and Inflammation in Parkinson Disease Pathology.” Journal of Neuroscience Research 96: 379–390. 10.1002/jnr.24185. 29072332

[brb370992-bib-0028] Kia, D. A. , D. Zhang , S. Guelfi , et al. 2021. “Identification of Candidate Parkinson Disease Genes by Integrating Genome‐Wide Association Study, Expression, and Epigenetic Data Sets.” JAMA Neurology 78: 464. 10.1001/jamaneurol.2020.5257. 33523105 PMC7851759

[brb370992-bib-0029] Kline, E. M. , M. C. Houser , M. K. Herrick , et al. 2021. “Genetic and Environmental Factors in Parkinson's Disease Converge on Immune Function and Inflammation.” Movement Disorders 36: 25–36. 10.1002/mds.28411. 33314312 PMC8285924

[brb370992-bib-0030] Kolbinger, F. , C. Huppertz , A. Mir , and F. D. Padova . 2016. “IL‐17A and Multiple Sclerosis: Signaling Pathways, Producing Cells and Target Cells in the Central Nervous System.” Current Drug Targets 17: 1882–1893. 10.2174/1389450117666160307144027. 26953244

[brb370992-bib-0031] Lang, Y. , F. Chu , D. Shen , et al. 2018. “Role of Inflammasomes in Neuroimmune and Neurodegenerative Diseases: A Systematic Review.” Mediators of Inflammation 2018: 1–11. 10.1155/2018/1549549. PMC5932495 29849483

[brb370992-bib-0032] Lee, S. W. , A. T. Vella , B. S. Kwon , and M. Croft . 2005. “Enhanced CD4 T Cell Responsiveness in the Absence of 4‐1BB.” Journal of Immunology 174: 6803–6808. 10.4049/jimmunol.174.11.6803. 15905521

[brb370992-bib-0033] Li, Q. , Z. Wang , H. Xing , Y. Wang , and Y. Guo . 2021. “Exosomes Derived From miR‐188‐3p‐Modified Adipose‐Derived Mesenchymal Stem Cells Protect Parkinson's Disease.” Molecular Therapy Nucleic Acids 23: 1334–1344. 10.1016/j.omtn.2021.01.022. 33717653 PMC7920810

[brb370992-bib-0034] Lin, J. , J.‐T. Li , L. Kong , et al. 2023. “Proinflammatory Phenotype in Major Depressive Disorder With Adulthood Adversity: In Line With Social Signal Transduction Theory of Depression.” Journal of Affective Disorders 341: 275–282. 10.1016/j.jad.2023.08.104. 37657624

[brb370992-bib-0035] Liu, D. , A. Baranova , and F. Zhang . 2024. “Evaluating the Causal Effect of Type 2 Diabetes on Alzheimer's Disease Using Large‐Scale Genetic Data.” Journal of Prevention of Alzheimer's Disease 11: 1280–1282. 10.14283/jpad.2024.148. 39350373

[brb370992-bib-0036] Liu, Z. , A.‐W. Qiu , Y. Huang , et al. 2019. “IL‐17A Exacerbates Neuroinflammation and Neurodegeneration by Activating Microglia in Rodent Models of Parkinson's Disease.” Brain, Behavior, and Immunity 81: 630–645. 10.1016/j.bbi.2019.07.026. 31351185

[brb370992-bib-0037] McGeachy, M. J. , D. J. Cua , and S. L. Gaffen . 2019. “The IL‐17 Family of Cytokines in Health and Disease.” Immunity 50: 892–906. 10.1016/j.immuni.2019.03.021. 30995505 PMC6474359

[brb370992-bib-0038] Mogi, M. , M. Harada , P. Riederer , H. Narabayashi , K. Fujita , and T. Nagatsu . 1994. “Tumor Necrosis Factor‐Alpha (TNF‐Alpha) Increases Both in the Brain and in the Cerebrospinal Fluid From Parkinsonian Patients.” Neuroscience Letters 165: 208–210. 10.1016/0304-3940(94)90746-3. 8015728

[brb370992-bib-0039] Nalls, M. A. , C. Blauwendraat , C. L. Vallerga , et al. 2019. “Identification of Novel Risk Loci, Causal Insights, and Heritable Risk for Parkinson's Disease: A Meta‐Analysis of Genome‐Wide Association Studies.” Lancet Neurology 18: 1091–1102. 10.1016/S1474-4422(19)30320-5. 31701892 PMC8422160

[brb370992-bib-0040] Parkinson, J. 2002. “An Essay on the Shaking Palsy.” Journal of Neuropsychiatry and Clinical Neurosciences 14: 223–236. 10.1176/jnp.14.2.223. 11983801

[brb370992-bib-0041] Patlola, S. R. , G. Donohoe , and D. P. McKernan . 2023. “The Relationship Between Inflammatory Biomarkers and Cognitive Dysfunction in Patients With Schizophrenia: A Systematic Review and Meta‐Analysis.” Progress in Neuro‐Psychopharmacology & Biological Psychiatry 121: 110668. 10.1016/j.pnpbp.2022.110668. 36283512

[brb370992-bib-0042] Qin, X. Y. , S. P. Zhang , C. Cao , Y. P. Loh , and Y. Cheng . 2016. “Aberrations in Peripheral Inflammatory Cytokine Levels in Parkinson Disease: a Systematic Review and Meta‐analysis.” JAMA Neurology 73: 1316. 10.1001/jamaneurol.2016.2742. 27668667

[brb370992-bib-0043] Reali, C. , M. Curto , V. Sogos , et al. 2003. “Expression of CD137 and Its Ligand in Human Neurons, Astrocytes, and Microglia: Modulation by FGF‐2.” Journal of Neuroscience Research 74: 67–73. 10.1002/jnr.10727. 13130507

[brb370992-bib-0044] Rhodes, S. L. , J. S. Sinsheimer , Y. Bordelon , J. M. Bronstein , and B. Ritz . 2011. “Replication of GWAS Associations for GAK and MAPT in Parkinson's Disease.” Annals of Human Genetics 75: 195–200. 21058943 10.1111/j.1469-1809.2010.00616.x PMC3074465

[brb370992-bib-0045] Sæther, L. S. , T. Ueland , B. Haatveit , et al. 2023. “Inflammation and Cognition in Severe Mental Illness: Patterns of Covariation and Subgroups.” Molecular Psychiatry 28: 1284–1292. 36577840 10.1038/s41380-022-01924-w PMC10005942

[brb370992-bib-0046] Sekula, P. , F. Del Greco M , C. Pattaro , and A. Köttgen . 2016. “Mendelian Randomization as an Approach to Assess Causality Using Observational Data.” Journal of the American Society of Nephrology 27: 3253–3265. 10.1681/ASN.2016010098. 27486138 PMC5084898

[brb370992-bib-0047] Servet‐Delprat, C. , S. Arnaud , P. Jurdic , et al. 2002. “Flt3+ Macrophage Precursors Commit Sequentially to Osteoclasts, Dendritic Cells and Microglia.” BMC Immunology 3: 15. 10.1186/1471-2172-3-15. 12398794 PMC134601

[brb370992-bib-0048] Shurin, M. 1998. “FLT3: Receptor and Ligand. Biology and Potential Clinical Application.” Cytokine & Growth Factor Reviews 9: 37–48. 10.1016/S1359-6101(97)00035-X. 9720755

[brb370992-bib-0049] Sun, W. , H. Cao , D. Liu , A. Baranova , F. Zhang , and X. Zhang . 2025. “Genetic Association and Drug Target Exploration of Inflammation‐Related Proteins With Risk of Major Depressive Disorder.” Progress in Neuro‐Psychopharmacology & Biological Psychiatry 136: 111165. 10.1016/j.pnpbp.2024.111165. 39383931

[brb370992-bib-0050] Tan, E.‐K. , Y.‐X. Chao , A. West , L.‐L. Chan , W. Poewe , and J. Jankovic . 2020. “Parkinson Disease and the Immune System—Associations, Mechanisms and Therapeutics.” Nature Reviews Neurology 16: 303–318. 10.1038/s41582-020-0344-4. 32332985

[brb370992-bib-0051] Xu, X. , D. Li , Q. He , J. Gao , B. Chen , and A. Xie . 2011. “Interleukin‐18 Promoter Polymorphisms and Risk of Parkinson's Disease in a Han Chinese Population.” Brain Research 1381: 90–94. 10.1016/j.brainres.2011.01.025. 21241672

[brb370992-bib-0052] Xu, Y. , W. Yue , Y. Y. Shugart , et al. 2016. “Potential Involvement of the Interleukin‐18 Pathway in Schizophrenia.” Journal of Psychiatric Research 74: 10–16. 10.1016/j.jpsychires.2015.12.013. 26736035

[brb370992-bib-0053] Yeo, Y. A. , J. M. Martínez Gómez , J. L. Croxford , S. Gasser , E.‐A. Ling , and H. Schwarz . 2012. “CD137 Ligand Activated Microglia Induces Oligodendrocyte Apoptosis via Reactive Oxygen Species.” Journal of Neuroinflammation 9: 173. 10.1186/1742-2094-9-173. 22799524 PMC3420242

[brb370992-bib-0054] Yi, M. , J. Li , S. Jian , et al. 2023. “Quantitative and Causal Analysis for Inflammatory Genes and the Risk of Parkinson's Disease.” Frontiers in Immunology 14: 1119315. 10.3389/fimmu.2023.1119315. 36926335 PMC10011457

[brb370992-bib-0055] Zhang, F. X. , and R. S. Xu . 2018. “Juglanin Ameliorates LPS‐Induced Neuroinflammation in Animal Models of Parkinson's Disease and Cell Culture via Inactivating TLR4/NF‐κB Pathway.” Biomedicine & Pharmacotherapy 97: 1011–1019. 10.1016/j.biopha.2017.08.132. 29136779

[brb370992-bib-0056] Zhang, Q. S. , Y. Heng , Y. H. Yuan , and N. H. Chen . 2017. “Pathological α‐Synuclein Exacerbates the Progression of Parkinson's Disease Through Microglial Activation.” Toxicology Letters 265: 30–37. 10.1016/j.toxlet.2016.11.002. 27865851

[brb370992-bib-0057] Zhao, J. H. , D. Stacey , N. Eriksson , et al. 2023. “Genetics of Circulating Inflammatory Proteins Identifies Drivers of Immune‐Mediated Disease Risk and Therapeutic Targets.” Nature Immunology 24: 1540–1551. 10.1038/s41590-023-01588-w. 37563310 PMC10457199

[brb370992-bib-0058] Zhao, Q. , A. Baranova , H. Cao , and F. Zhang . 2024. “Gut Microbiome and Major Depressive Disorder: Insights From Two‐Sample Mendelian Randomization.” BMC Psychiatry 24: 493. 10.1186/s12888-024-05942-6. 38977973 PMC11232322

[brb370992-bib-0059] Zhao, Q. , D. Liu , A. Baranova , H. Cao , and F. Zhang . 2024. “Novel Insights into the Causal Effects and Shared Genetics Between Body Fat and Parkinson Disease.” CNS Neuroscience & Therapeutics 30: e70132. 10.1111/cns.70132. 39578713 PMC11584348

[brb370992-bib-0060] Zhao, Y. , X. Zhang , N. Guo , et al. 2022. “Genetically Predicted Levels of Circulating Inflammatory Cytokines and the Risk and Age at Onset of Parkinson's Disease: A Two‐Sample Mendelian Randomization Study.” Frontiers in Aging Neuroscience 14: 811059. 10.3389/fnagi.2022.811059. 35299944 PMC8923644

[brb370992-bib-0061] Zheng, T. , T. Jiang , R. Li , Y. Zhu , Q. Han , and M. Wang . 2024. “Circulating Interleukins Concentrations and Post‐Stroke Depression: A Systematic Review and Meta‐Analysis.” Progress in Neuro‐Psychopharmacology & Biological Psychiatry 134: 111050. 10.1016/j.pnpbp.2024.111050. 38844127

[brb370992-bib-0062] Zou, L. , Y. Tian , Y. Wang , et al. 2023. “High‐Cholesterol Diet Promotes Depression‐ and Anxiety‐Like Behaviors in Mice by Impact Gut Microbe and Neuroinflammation.” Journal of Affective Disorders 327: 425–438. 10.1016/j.jad.2023.01.122. 36738999

